# Predicted transmembrane proteins with homology to Mef(A) are not responsible for complementing *mef*(A) deletion in the *mef*(A)–*msr*(D) macrolide efflux system in *Streptococcus pneumoniae*

**DOI:** 10.1186/s13104-021-05856-6

**Published:** 2021-11-25

**Authors:** Valeria Fox, Francesco Santoro, Gianni Pozzi, Francesco Iannelli

**Affiliations:** Department of Medical Biotechnologies, University of Siena, Siena, Italy

**Keywords:** *Mef*(A), *Msr*(D), Macrolide efflux, *Streptococcus pyogenes*, *Streptococcus pneumoniae*, ABC-transporter, ϕ1207.3, Prophage

## Abstract

**Objectives:**

In streptococci, the type M resistance to macrolides is due to the *mef*(A)–*msr*(D) efflux transport system of the ATP-Binding cassette (ABC) superfamily, where it is proposed that *mef*(A) codes for the transmembrane channel and *msr*(D) for the two ATP-binding domains. Phage ϕ1207.3 of *Streptococcus pyogenes*, carrying the *mef*(A)–*msr*(D) gene pair, is able to transfer the macrolide efflux phenotype to *Streptococcus pneumoniae*. Deletion of *mef*(A) in pneumococcal ϕ1207.3-carrying strains did not affect erythromycin efflux. In order to identify candidate genes likely involved in complementation of *mef(*A) deletion, the Mef(A) amino acid sequence was used as probe for database searching.

**Results:**

In silico analysis identified 3 putative candidates in the *S. pneumoniae* R6 genome, namely *spr0971*, *spr1023* and *spr1932*. Isogenic deletion mutants of each candidate gene were constructed and used in erythromycin sensitivity assays to investigate their contribution to *mef*(A) complementation. Since no change in erythromycin sensitivity was observed compared to the parental strain, we produced double and triple mutants to assess the potential synergic activity of the selected genes. Also these mutants did not complement the *mef*(A) function.

**Supplementary Information:**

The online version contains supplementary material available at 10.1186/s13104-021-05856-6.

## Introduction

Macrolide resistance in streptococci is usually associated with two major mechanisms: (i) target-site modification, mediated by the erythromycin ribosomal methylase (*erm*) family genes responsible for 23S rRNA methylation; (ii) active drug efflux, mediated by the *mef* family genes which confer the M phenotype, characterized by low level resistance to 14- and 15-membered macrolides [1–6]. The two *mef* major allelic variants, *mef*(A) and *mef*(E), were originally described in *Streptococcus pyogenes* and in *Streptococcus pneumoniae*, respectively [7, 8]. These variants are highly homologous and are also found in other streptococcal species, gram-positive and gram-negative genera [3, 5, 9–12] (for an updated list see the Dr. Marylin Roberts’s website https://faculty.washington.edu/marilynr). The *mef* alleles are associated to different chromosomal genetic elements. In *S. pneumoniae*, we found Tn*1207.1*, a 7244-bp non-conjugative element carrying *mef*(A), whereas the 5532-bp pneumococcal genetic element (mega) was found to carry *mef*(E) [13–17]. In *S. pyogenes*, we described the 52,491-bp prophage Φ1207.3 carrying *mef*(A) whose left 7244-bp sequence is identical to Tn*1207.1* [18–20]. In clinical isolates of *S. pyogenes* other *mef*(A)-carrying prophages were found, including Φ10394.4, Φm46.1 and its variant VP_00501.1 [21–23]. In the *mef*-carrying genetic elements, the *msr*(D) gene was always associated to and co-transcribed with the *mef* gene and contributes to macrolide efflux resistance [23–28]. In our previous work, genome database search showed that in 33 out of 37 genomes, *mef*(A) was associated in tandem to *msr*(D), while bioinformatic analysis showed that the Mef(A) protein was predicted to form six transmembrane helices and the Msr(D) protein to have two Nucleotide Binding Domains (NBDs) typical of ATP-binding transporters [27]. We hypothesized that *mef(*A) and *msr*(D) constitute a two-gene ATP-Binding Cassette efflux transport system, where *mef*(A) encodes the transmembrane channel, and *msr(*D) the two ATP-binding domains. A functional analysis of the relative contribution of *mef*(A) and *msr*(D) to macrolide resistance supported this hypothesis, showing that deletion of *msr*(D) abolishes erythromycin resistance, whereas deletion of *mef*(A) causes only a twofold reduction of MIC value [27]. It is likely that in absence of Mef(A), Msr(D) utilizes an alternative transmembrane channel for macrolide efflux. In the present work, a pneumococcal genome homology search was used to investigate the presence of transmembrane proteins homologous to Mef(A), which could complement the Mef(A) function. Three genes encoding transmembrane proteins were identified and their role as alternative Msr(D) cognate transmembrane channel was investigated through site specific mutagenesis and functional studies.

## Main text

### Methods

#### Bacterial strains, growth and mating condition

All pneumococcal strains used in this work and their relevant properties are reported in Table 1. Bacterial strains were grow grown in Tryptic Soy Broth (TSB) or Tryptic Soy Agar (TSA) supplemented with 3% defibrinated horse blood [29]. Transfer of Φ1207.3 or Φ1207.3Δ*mef*(A) from strains FR183 and FP40 to the deletion mutants was obtained through a mating protocol as already reported [30]. Briefly, donor and recipient cells were grown separately in TSB in the presence of the appropriate antibiotics. Upon reaching the end of exponential phase, cells were mixed at a donor-recipient 1:10 ratio, centrifuged, and pellet was plated on TSA plates supplemented with 5% blood. Plates were incubated at 37 °C in the presence of 5% CO_2_ for 4 h and cells were recovered with a cotton swab and resuspended in TSB. To select for recombinants, cell suspension was plated following a multilayer plating procedure [30].

**Table 1:** Bacterial strains

Strain	Properties^a^	References
Rx1	Unencapsulated D39 *S. pneumoniae* derivative	[36, 44]
DP1004	Rx1 derivative, *str-41*, Sm^R^	[29, 45]
FR183	DP1004 derivative, carrying φ1207.3, Sm^R^, Em^R^	[27]
FP40	FR183 derivative, carrying φ1207.3Δ*mef*(A), Sm^R^, Em^R^, Cm^R^	[27]
FR323	DP1004 derivative, *spr0971*ΔSpe, Sm^R^, Spe^R^	This study
FR324	DP1004 derivative, *spr1023*ΔKm, Sm^R^, Km^R^	This study
FR325	DP1004 derivative, *spr1932*ΔKm, Sm^R^, Km^R^	This study
FR358	FR323 derivative, carrying φ1207.3, Sm^R^, Em^R^, Spe^R^	This study
FR359	FR324 derivative, carrying φ1207.3, Sm^R^, Em^R^, Spe^R^	This study
FR360	FR325 derivative, carrying φ1207.3, Sm^R^, Em^R^, Spe^R^	This study
FR355	FR323 derivative, carrying φ1207.3Δ*mef*(A), Sm^R^,Em^R^, Cm^R^, Spe^R^	This study
FR356	FR324 derivative, carrying φ1207.3Δ*mef*(A), Sm^R^, Em^R^, Cm^R^, Spe^R^	This study
FR357	FR325 derivative, carrying φ1207.3Δ*mef*(A), Sm^R^, Em^R^, Cm^R^, Spe^R^	This study
FR335	FR323 derivative, *spr0971*ΔSpe, *spr1023*ΔKm, Sm^R^, Spe^R^, Km^R^	This study
FR336	FR323 derivative, *spr0971*ΔSpe, *spr1932*ΔKm, Sm^R^, Spe^R^, Km^R^	This study
FR337	FR325 derivative, *spr1932*ΔKm, Δ*spr1023* (*in-frame*), Sm^R^, Km^R^	This study
FR344	FR335 derivative, carrying φ1207.3, Sm^R^, Em^R^, Spe^R^, Km^R^	This study
FR345	FR336 derivative, carrying φ1207.3, Sm^R^, Em^R^, Spe^R^, Km^R^	This study
FR349	FR337 derivative, carrying φ1207.3, Sm^R^, Em^R^, Km^R^	This study
FR346	FR335 derivative, carrying φ1207.3Δ*mef*(A), Sm^R^, Em^R^, Cm^R^, Spe^R^, Km^R^	This study
FR347	FR336 derivative, carrying φ1207.3Δ*mef*(A), Sm^R^, Em^R^, Cm^R^, Spe^R^, Km^R^	This study
FR348	FR337 derivative, carrying φ1207.3Δ*mef*(A), Sm^R^, Em^R^, Cm^R^, Km^R^	This study
FR338	FR337 derivative, *spr*1932ΔKm, Δ*spr*1023 (*in-frame*), *spr*0971ΔSpe, Sm^R^, Km^R^, Spe^R^	This study
FR351	FR338 derivative, carrying φ1207.3, Sm^R^, Em^R^, Km^R^, Spe^R^	This study
FR350	FR338 derivative, carrying φ1207.3Δ*mef*(A), Sm^R^, Em^R^, Cm^R^, Km^R^, Spe^R^	This study

*Sm* streptomycin, *Em* erythromycin, *Cm* chloramphenicol, *Spe* spectinomycin, *Km* kanamycin

^a^*str-41* indicates a point mutation conferring resistance to streptomycin

#### Bioinformatic analysis

Homology searches of the pneumococcal genome R6 available at the National Center for Biotechnology Information (https://www.ncbi.nlm.nih.gov/genome/microbes/) was performed using Microbial BLAST with the Megablast algorithm (https://blast.ncbi.nlm.nih.gov/Blast.cgi?PROGRAM=blastp&PAGE_TYPE=BlastSearch&BLAST_SPEC=MicrobialGenomes&LINK_LOC=blasttab&LAST_PAGE=blastp). Default parameters were used and only alignments with significant e-values (< 0.001) were considered. Protein sequence analysis was carried out with the softwares TMpred and Phyre2 [31–34].

#### Gene SOEing PCR mutagenesis

Isogenic deletion mutants were obtained transforming *S. pneumoniae* Rx1 derivative recipients with mutagenic constructs assembled by Gene Splicing by Overlap Extension (Gene SOEing) [29]. The oligonucleotide primers used for mutagenesis, sequencing and PCR selection of the recombinants strain are reported in Additional file 1: Table S1. Deletion of *spr0971* coding sequence (CDS) was obtained with a mutagenic construct containing the *ami/aad9* spectinomycin resistance cassette (894 bp) [35] flanked by the upstream (601 bp) and downstream (459 bp) *spr0971* flanking fragments, respectively. The *spr1023* CDS was deleted with a mutagenic construct containing the *ami/aphIII* kanamycin resistance cassette (1033 bp) [36] joined to the left (696 bp) and right (658 bp) *spr1023* flanking fragments. The *spr1932* mutagenic construct contained the kanamycin resistance cassette flanked by the upstream (724 bp) and downstream (694 bp) *spr1932* flanking fragments. The mutagenic construct for *spr1023* in-frame deletion was obtained assembling the DNA fragments located upstream (749 bp) and downstream (773 bp) of *spr1023* CDS. Linear PCR constructs were used directly as donor DNA in transformation experiments. Recombinant strains were selected for acquisition of spectinomycin or kanamycin resistance, while deletion of *spr1023* was selected by selective PCR analysis [29]. The correct integration of constructs was confirmed by PCR and sequencing.

#### Minimal inhibitory concentration (MIC) determination

The minimal inhibitory concentration (MIC) was assessed by microdilution method, according to the Clinical and Laboratory Standards Institute guideline (CLSI, 2020) as already reported [27]. Briefly, bacteria were grown in TSB until reaching the exponential phase (OD_590_ = 0.3, corresponding to approximately 10^8^ CFU/ml), then culture aliquots were taken and frozen at − 70 °C in 10% glycerol. Frozen cultures were then thawed, diluted 1:100 in TSB (10^6^ CFU/ml) and 100 µl were added to a 96-wells microplate containing 100 µl of serial twofold dilutions of erythromycin, reaching a final concentration of 5 × 10^5^ CFU/ml in each well. Plates were incubated at 37 °C and visually analyzed after 18 h. Bacterial growth was assessed using the microplate ELISA reader VERSAmax (Molecular Devices). The *S. pneumoniae* ATCC49619 strain was used as a quality control. MIC assays were performed in quintuplicate with at least two technical replicates per experiment.

### Results

#### Identification and sequence analysis of a candidate genes encoding a Mef(A) homologous protein

The 405-aa Mef(A) sequence (GenBank accession no. AAT72347) was used as a query to conduct a BLAST homology search of *S. pneumoniae* R6 genome. Homology analysis revealed the presence of three genes coding for proteins with a significant homology (e-value < 0.001) to Mef(A): (i) *spr0971* (GenBank accession number NP_358565.1); (ii) *spr1023* (GenBank accession number NP_358617.1); (iii) *spr1932* (GenBank accession number NP_359523.1). The *spr0971* gene, annotated as “ABC transporter membrane-spanning permease—macrolide efflux”, codes for a 403 aa protein displaying 23% identity to Mef(A). The *spr1023* gene, annotated as “macrolide ABC transporter permease”, codes for a 392 aa protein with 24% identity to Mef(A). The *spr1932* gene, annotated as “hypothetical protein”, codes for a 415 aa protein with 21% identity to Mef(A). Analysis of the transmembrane domains of all deduced amino acid products predicted the presence of up to 12 transmembrane helices.

#### Investigation of the role of the candidate genes on Mef(A) complementation

**Fig. 1:**
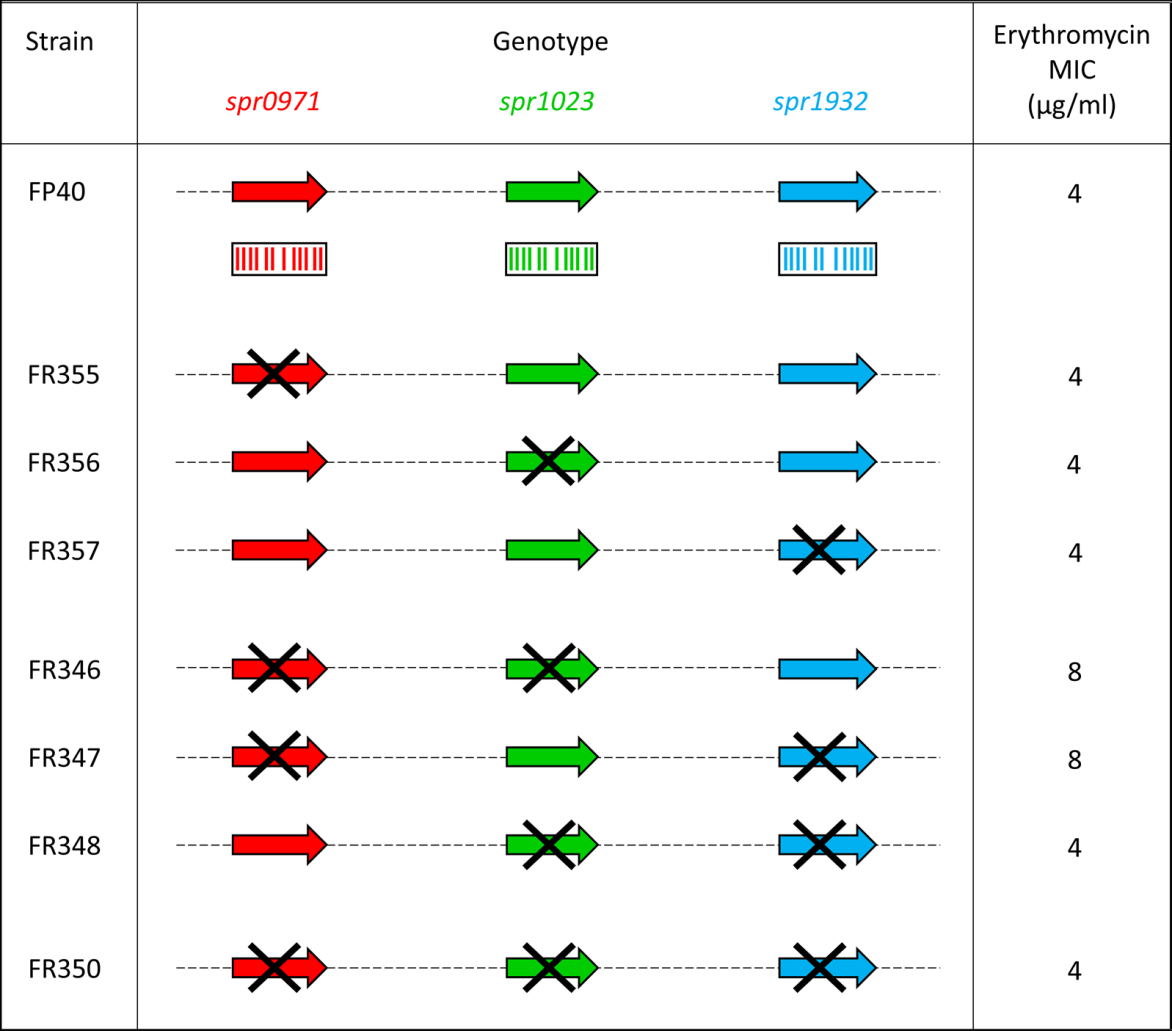
Schematic representation of genotype and phenotype of *S. pneumoniae* isogenic deletion mutants. Macrolide efflux in Streptococci is associated to the *mef*(A)–*msr*(D) macrolide efflux system. The contribution of *spr0971*, *spr1023*, *spr1932* genes to *mef*(A) complementation was investigated by constructing isogenic deletion mutants, carrying the recombinant Φ1207.3Δ*mef*(A) phage. Single, double and triple mutants were obtained by PCR gene SOEing and transformation. Genes are reported as arrows, while the cross indicates gene deletions. Spr0971, Spr1023 and Spr1932 proteins are reported as not scaled boxes and the 12 transmembrane domains as bars

To define if the selected candidate genes could complement the *mef*(A) function, we constructed three isogenic deletion mutants in *S. pneumoniae* DP1004 background (Table 1). The 1209 bp *spr0971* CDS was deleted and replaced by the 894-bp *ami/aad9* cassette, whereas the 1176 bp *spr1023* and the 1245 bp *spr1932* CDSs were deleted by allelic replacement with the 1033-bp *ami/aphIII* cassette. These mutants were used as recipients to obtain derivative strains harboring the recombinant Φ1207.3Δ*mef*(A). Sensitivity to erythromycin of the isogenic mutants was assessed by determining the MIC. In our previous study, we reported an erythromycin MIC of 4 µg/ml for the Φ1207.3Δ*mef*(A)-carrying strain FP40, consistent with the presence of an alternative transmembrane channel able to complement the *mef*(A) function [27]. Deletion of *spr0971, spr1023* and *spr1932*, did not affect erythromycin sensitivity (Fig. 1 and Table 2). Then, to investigate if the Mef(A) complementation is due to a synergic action of these genes, we constructed double deletion mutants. The *spr0971-spr1023* and *spr0971-spr1932* double deletion mutants were obtained transforming strain FR323 (*spr0971*ΔSpe) with the *spr1023* and *spr1932* kanamycin mutagenic constructs, respectively, while the *spr1023*–*spr1932* double mutant was obtained by transforming the strain FR325 (*spr1932*ΔKm) with a mutagenic construct designed to produce *spr1023* in frame deletion. Deletion of *spr1023* and *spr1932*, did not affect erythromycin sensitivity, whereas the *spr0971*–*spr1023* and *spr0971*–*spr1932* deletions produce a twofold increase of erythromycin MIC (8 µg/ml). Finally, a triple *spr0971*–*spr1023*–*spr1932* mutant was constructed using the *spr1023*–*spr1932* double mutant FR337 as recipient and the *spr0971* spectinomycin mutagenic construct as donor DNA. No change in erythromycin sensitivity (MIC = 4 µg/ml) was observed also for the triple mutant. As control strains we used: (i) the parental strain DP1004; (ii) strain FR183 carrying the Φ1207.3 phage; (iii) strain FP40 carrying the Φ1207.3Δ*mef*(A) recombinant phage; (iv) deletion mutants without Φ1207.3; (v) deletion mutants carrying Φ1207.3.

**Table 2:** Erythromycin sensitivity

Strain	Genotype					Phenotype^b^	Erythromycin MIC (µg/ml)
	Chromosome			Φ1207.3^a^			
	*spr0971*	*spr1023*	*spr1932*	*mef*(A)	*msr(D)*		
DP1004	+	+	+	np	np	Sensitive	0.06
FR183	+	+	+	+	+	Resistant	8
FP40	+	+	+	−	+	Resistant	4
FR323	−	+	+	np	np	Sensitive	0.06
FR324	+	−	+	np	np	Sensitive	0.06
FR325	+	+	−	np	np	Sensitive	0.06
FR358	−	+	+	+	+	Resistant	8
FR359	+	−	+	+	+	Resistant	8
FR360	+	+	−	+	+	Resistant	8
FR355	−	+	+	−	+	Resistant	4
FR356	+	−	+	−	+	Resistant	4
FR357	+	+	−	−	+	Resistant	4
FR335	−	−	+	np	np	Sensitive	0.06
FR336	−	+	−	np	np	Sensitive	0.06
FR337	+	−	−	np	np	Sensitive	0.06
FR344	−	−	+	+	+	Resistant	8
FR345	−	+	−	+	+	Resistant	8
FR349	+	−	−	+	+	Resistant	8
FR346	−	−	+	−	+	Resistant	8
FR347	−	+	−	−	+	Resistant	8
FR348	+	−	−	−	+	Resistant	4
FR338	−	−	−	np	np	Sensitive	0.06
FR351	−	−	−	+	+	Resistant	8
FR350	−	−	−	−	+	Resistant	4

^a^np: Φ1207.3 phage not present

^b^MIC interpretative standards: sensitive ≤ 0.25 µg/ml, intermediate = 0.5 µg/ml, and resistant ≥ 1 µg/ml

### Conclusions

Our previous findings and those of other research groups [23–28] reported that the macrolide efflux in Streptococci relies on the presence of the *mef(*A)–*msr*(D) operon. Based on bioinformatic analysis of the Mef(A) and Msr(D) proteins and their functional characterization, we proposed that *mef*(A) and *msr*(D) constitute a two-gene ATP-Binding Cassette efflux transport system, where *mef*(A) encodes the transmembrane channel, and *msr*(D) the two ATP-binding domains. Msr(D) is a member of the ACB-F family of ABC transporters, which are widespread among both bacteria and eukaryotes [37]. In contrast to other members of this family, which were shown to interact with the ribosome and protect it from antibiotics [38, 39], Msr(D) (i) is always associated to a cognate transmembrane domain encoded by the *mef*(A) gene, (ii) was shown to be involved in the erythromycin efflux from the bacterial cell [27] and (iii) was shown to be associated to Mef(A) and localized on the membrane [25].

Since deletion of *msr*(D) abolishes erythromycin resistance, whereas deletion of *mef*(A) causes only a twofold reduction of MIC value, we hypothesized that in absence of Mef(A), Msr(D) recruits an alternative transmembrane partner. In this work, to determine if the *mef*(A) deletion is complemented by pneumococcal chromosomal genes, we investigated the presence of genes encoding transmembrane proteins homologous to Mef(A). Homology search identified three candidate genes, namely *spr0971*, *spr1023*, *spr1932*. Isogenic single, double and triple deletion mutants were constructed and the single and synergic contribution of these genes to *mef*(A) complementation was assessed by erythromycin sensitivity assays. The expected decrease of erythromycin MIC, due to the absence of a putative alternative Mef(A) channel, was not observed, suggesting that these genes are not involved in the complementation of *mef*(A) deletion. For the two *spr0971*–*spr1023* and *spr0971*–*spr1932* double mutants, we observed a twofold increase of the erythromycin MIC, which was not seen in the triple mutant. These results are unexpected, as the MIC value in absence of *mef*(A) and other alternative transmembrane channels would be predicted to decrease. The same increase was observed also following the deletion of a fourth gene, *spr0875* (data not shown). This gene, which encodes a protein homologous to Mef(A), was previously characterized and associated to the efflux of other compounds including fusidic acid and sodium dodecyl sulfate [40, 41]. For this reason and because its deletion resulted in the increase of erythromycin MIC value, we excluded it from further investigations. We hypothesise that this increase could be due a possible “unspecific” permease activity which allows erythromycin entrance in the bacterial cell through one or more of the pores encoded by these genes. In conclusion the quest to identify the alternative Msr(D) cognate transmembrane channel remains open.

## Limitations

Investigation of putative candidate genes, responsible for complementing *mef*(A) deletion in the *mef*(A)–*msr*(D) macrolide efflux system in *S. pneumoniae*, was performed using a targeted approach based on the homology to Mef(A). Nonetheless, it is possible that the proteins involved in this complementation may not display significant homology to Mef(A). A genome-wide approach based on the creation and screening of a library of random mariner transposon mutants [42, 43], allowing for random mutagenesis of the whole pneumococcal genome, could be used to investigate the possible effect of other chromosomal genes in *mef*(A) complementation.

## Supplementary Information

**Additional file 1: Table S1.** Oligonucleotides primer used to construct the isogenic deletion mutants.

## Data Availability

All data generated or analysed during this study are included in this published article. The pneumococcal genome R6 is available at the National Center for Biotechnology Information (https://www.ncbi.nlm.nih.gov/genome/microbes/) under accession number AE007317.1. Protein search was performed with Microbial BLAST using the Megablast algorithm (https://blast.ncbi.nlm.nih.gov/Blast.cgi?PROGRAM=blastp&PAGE_TYPE=BlastSearch&BLAST_SPEC=MicrobialGenomes&LINK_LOC=blasttab&LAST_PAGE=blastp). The Mef(A) protein sequence is available at the GenBank accession no. AAT72347; *spr0971*, *spr1023*, and *spr1932* protein sequences are available under accession numbers NP_358565.1, NP_358617.1, and NP_359523.1, respectively.

## Abbreviations

ABC: ATP-Binding cassette
erm: Erythromycin ribosomal methylase
Gene SOEing: Gene Splicing by Overlap Extension
NBDs: Nucleotide Binding Domains
MIC: Minimal inhibitory concentration
TSB: Tryptic Soy Broth
TSA: Tryptic Soy Agar
