# Supplementary material for: Predicted transmembrane proteins with homology to Mef(A) are not responsible for complementing mef(A) deletion in the mef(A)–msr(D) macrolide efflux system in Streptococcus pneumoniae
Source: BMC Res Notes. 2021 Nov 25;14:432. doi: 10.1186/s13104-021-05856-6 (PMC8620141; doi:10.1186/s13104-021-05856-6)
Supplement: Supplementary file 1 — Additional file 1: Table S1. Oligonucleotides primer used to construct the isogenic deletion mutants. [file 13104_2021_5856_MOESM1_ESM.docx]

**Table S1.** Oligonucleotides primer used to construct the isogenic deletion mutants

| **Name** | **Sequence (5’to 3’)** | **Target** | **Notes** | **GenBank ID: nucleotides** |
| --- | --- | --- | --- | --- |
| IF100 | GCTCTAGAACTAGTGGATC | Spectinomycin resistance cassette, 5’ end |  | AY334020: 1-16 |
| IF101 | TTCCCTTCAAGAGCGATAC | Spectinomycin resistance cassette, 3’ end |  | AY334020: 890-872 |
| IF149 | CAAGCTGGGGATCCGTTTGAT | Kanamycin resistance cassette, 5’ end |  | AY334018: 5-25 |
| IF190 | GATACAAATTCCTCGTAGG | Kanamycin resistance cassette, 3’ end |  | AY334019.1: 1009-1027 |
| IF1077 | CACTTGTAGAAATCGGTGACA | *spr*0971 upstream fragment, 5’ end |  | AE007317.1: 952717-952737 |
| IF1078 | GGATCCACTAGTTCTAGAGCGATCCTCATCTTAGTTCATTCA | *spr*0971 upstream fragment, 3’end | the first 20 nucleotides are complementary to IF100 | AE007317.1: 953296-953317 |
| IF1079 | GTATCGCTCTTGAAGGGAATGAATGTGAAGGGCAATCTGTT | *spr*0971 downstream fragment,5’ end | the first 19 nucleotides are complementary to IF101 | AE007317.1: 954651-954672 |
| IF1080 | GGTCAAAATCTATTAACCGTCT | *spr*0971 downstream fragment, 3’ end |  | AE007317.1: 955088-955109 |
| IF1081 | CATGTGTCTATGATGGCACAG | *spr*1023 upstream fragment, 5’ end |  | AE007317.1: 1001488-1001508 |
| IF1082 | AATCAAACGGATCCCCAGCTTGGTATCAAAACCGAAAGGAGC | *spr*1023 upstream fragment, 3’ end | the first 22 nucleotides are complementary to IF149 | AE007317.1:1002164-1002183 |
| IF1083 | CCTACGAGGAATTTGTATCCGCCCTTCTCGTTTGTGAGGA | *spr*1023 downstream fragment, 5’ end | the first 19 nucleotides are complementary to IF190 | AE007317.1:1003438-1003458 |
| IF1084 | CTGTATCCAACTGACGCAGAGT | *spr*1023 downstream fragment, 3’ end |  | AE007317.1: 1004074-1004095 |
| IF1085 | TAGCACCTCTTGAGGACTTA | *spr*1932 upstream fragment, 5’ end |  | AE007317.1: 1915042-1915061 |
| IF1086 | ATCAAACGGATCCCCAGCTTGGCACTTATGAATTTGCGAAACG | *spr*1932 upstream fragment, 3’ end | the first 21 nucleotides are complementary to IF149 | AE007317.1:1914338-1914359 |
| IF1087 | CCTACGAGGAATTTGTATCCTGTTTTAATCCCTTTATTTGTGG | *spr*1932 downstream fragment, 5’ end | the first 19 nucleotides are complementary to IF190 | AE007317.1:1912996-1913019 |
| IF1088 | TCCTTAGAGTCCAAGACACGAAG | *spr*1932 downstream fragment, 3’ end |  | AE007317.1: 1912326-1912348 |
| IF1097 | GACAAGATGATAACACCACTTCATTAAAGCTCCTCTCAAAT | *spr*1023 upstream fragment, 3’ end | the first 22 nucleotides are complementary to IF1099 | AE007317.1:1002196-1002214 |
| IF1099 | GAAGTGGTGTTATCATCTTGTC | *spr*1023 downstream fragment, 5’ end |  | AE007317.1: 1003323- 1003344 |
